# Exploring Non-Gaussianity Reduction in Quantum Channels

**DOI:** 10.3390/e27070768

**Published:** 2025-07-20

**Authors:** Micael Andrade Dias, Francisco Marcos de Assis

**Affiliations:** 1 QuIIN—Quantum Industrial Innovation, EMBRAPII CIMATEC Competence Center in Quantum Technologies, SENAI CIMATEC, Av. Orlando Gomes 1845, Salvador 41650-010, BA, Brazil; 2 Department of Electrical and Photonics Engineering, Technical University of Denmark, 2800 Lyngby, Denmark; 3 Department of Electrical Engineering, Federal University of Campina Grande, Campina Grande 58429-900, PB, Brazil; fmarcos@dee.ufcg.edu.br

**Keywords:** quantum resource theory, Gaussian channels, non-Gaussianity

## Abstract

The quantum relative entropy between a quantum state and its Gaussian equivalent is a quantifying function of the system’s non-Gaussianity, a useful resource in several applications, such as quantum communication and computation. One of its most fundamental properties is to be monotonically decreasing under Gaussian evolutions. In this paper, we develop the conditions for a non-Gaussian quantum channel to preserve the monotonically decreasing property. We propose a necessary condition to classify between Gaussian and non-Gaussian channels and use it to define a class of quantum channels that decrease the system’s non-Gaussianity. We also discuss how this property, combined with a restriction on the states at the channel’s input, can be applied to the security analysis of continuous-variable quantum key distribution protocols.

## 1. Introduction

Quantum resource theory (QRT) stands as a cornerstone in the field of quantum information science, providing a formal framework for understanding and manipulating various quantum resources [1]. From entanglement to coherence, quantum resource theories offer a systematic approach to quantifying and harnessing the unique properties of quantum systems, allowing advancements in quantum communication, computation, and beyond [2,3].

A non-Gaussian (nG) resource theory, for instance, aims to understand which tasks require states and operations to have an nG character and how they can be better used, once the Gaussian sector of quantum states and operations is simpler to manipulate in a laboratory. Central to the study of an nG quantum resource theory (nG-QRT) is the quantum relative entropy (QRE), a powerful tool for quantifying the non-Gaussianity of quantum states [4]. In particular, the QRE plays a pivotal role as a resource quantifying function, offering insights into the distinguishability between a quantum state and its Gaussian equivalent [5,6]. A key property of the quantum relative entropy is its monotonic decrease under Gaussian operations, providing a robust foundation for characterizing nG transformations and their effects on the system’s non-Gaussianity.

Although quantum resource theory has found widespread applications across various domains of quantum information science [7,8,9,10], its integration into continuous-variable quantum key distribution (CV-QKD) protocols has been relatively limited. In some foundational studies, its developments have been utilized merely to corroborate well-known results from the existing literature, such as the optimality of Gaussian states for entropic quantities, rather than being actively exploited to improve the capabilities and security of CV-QKD schemes [11]. However, one promising avenue for application lies in the security analysis of CV-QKD protocols that utilize nG modulation of coherent states.

In the general setup of a QKD protocol, Alice and Bob (the trusted parties) distribute secret random keys by transmitting quantum states through an untrusted quantum channel. An eavesdropper (also called Eve) has access to the quantum channel and attempts to gain information employing some attack strategy. So, to keep secrecy, Alice and Bob must estimate how much information Eve has had access to during the protocol execution. When assessing this quantity, Alice and Bob must decide which model they will use to describe the quantum channel linking them, either a Gaussian or an nG. When assuming a Gaussian model, they in fact can upper bound Eve’s information by reconstructing a covariance matrix using solely the channel transmittance and excess noise parameters. Despite its practical relevance, the Gaussian channel model does not cover the worst-case scenario of Eve’s attacks, given that Alice did not prepare her states according to a Gaussian distribution [12].

State-of-the-art security analyses of CV-QKD with nG modulation often involve sophisticated optimization techniques [12,13,14]. These analyses aim to determine the maximal eavesdropper information by exploring the space of nG quantum channels compatible with the estimated parameters during quantum communication. When nG modulation is used for quantum state transmission, the non-Gaussianity must be taken into account in the security analysis.

In this paper, we investigate the gap between the Gaussian and nG security models of CV-QKD by using tools from nG-QRT. More precisely, we take as starting point one basic property of the QRE measure of non-Gaussianity, its monotone decrease under Gaussian operations, and investigate how it can be extended to nG quantum channels. By presenting the conditions under which an nG quantum channel reduces the system’s non-Gaussianity, we discuss how it can be used in security proofs of CV-QKD protocols with nG modulation. We also provide examples of such quantum channels, showing that, for specific mixtures of quantum states at the channel input, the covariance matrix remains unchanged while the system nG is reduced.

The remainder of the paper is structured as follows: Section 2 states the formal definitions and the problem we aim to address. In Section 3, we develop the main results for nG quantum channels, and in Section 4, we explore how it can be used in the security analysis of CV-QKD protocols, with the concluding remarks in Section 5.

### Notation

In what follows, we use the standard Dirac notation for quantum mechanics. *A* and *B* are quantum systems with the associated Hilbert spaces $H_{A}$ and $H_{B}$, respectively. $B(H_{A})$ and $D(H_{A})$ denote the space of bounded linear operators and the set of density operators in $H_{A}$, respectively, with elements represented as $\hat{A}\in B(H_{A})$ and $\hat{\rho }\in D(H_{A})$. The subset of $D(H_{A})$ corresponding to Gaussian states will be denoted by $G_{s}\left(H_{A}\right)$, or simply $G_{s}$.

The subspace of completely positive trace-preserving (CPTP) linear operators from $H_{A}$ to $H_{B}$ is denoted as $Q(H_{A}\to H_{B})$ with elements $N_{A\to B}$. All indexes will be dropped when implicit. If N is a quantum channel, the evolution of a state and the transformations of any of its quantities are represented by N(·) or $\overset{N}{\to }$. In particular, we denote by $G_{c}\left(H_{A}\to H_{B}\right)\subset Q\left(H_{A}\to H_{B}\right)$ the subset of all Gaussian quantum channels, or only $G_{c}$.

## 2. Preliminaries and Problem Statement

The question we are proposing is as follows: Can a quantum state have its non-Gaussianity reduced after undergoing an nG evolution? Alternatively, is there any nG channel that makes quantum states “more Gaussian”? By non-Gaussianity of an arbitrary quantum state, the literature often refers to a quantity informing “how much” an nG state fails to pass as a Gaussian state. Operationally, it may be defined as the distance from a Gaussian reference state [4]. Here, we use the QRE as a quantifying function of non-Gaussianity, having in mind that, even though it is not a metric, it satisfies all axioms for a resource quantifying function [1].

**Definition** **1** ([6])**.** *Let $\hat{\sigma }$ be an arbitrary quantum state and ${\hat{\sigma }}^{G}$ be the Gaussian quantum state with the same mean vector and covariance matrix as $\hat{\sigma }$. The state ${\hat{\sigma }}^{G}$ is said to be the Gaussian equivalent to $\hat{\sigma }$. The QRE-based nG measure of $\hat{\sigma }$ is defined as*

(1) $$\delta_{vN}\left(\hat{\sigma }\right)=S(\hat{\sigma }||{\hat{\sigma }}^{G}).$$

Among the many properties of $\delta_{vN}$, two of them are of special interest:

(i) Non-negativity ($\delta_{vN}\left(\hat{\sigma }\right)\geq 0$, with equality if and only if $\hat{\sigma }\in G_{s}$);
(ii) Contractivity under Gaussian quantum channels, $\delta_{vN}\left(\hat{\sigma }\right)\geq \delta_{vN}\left(N\left(\hat{\sigma }\right)\right)$ for any $\hat{\sigma }\in D(H)$ and $N\in G_{c}$.

**Figure 1:**
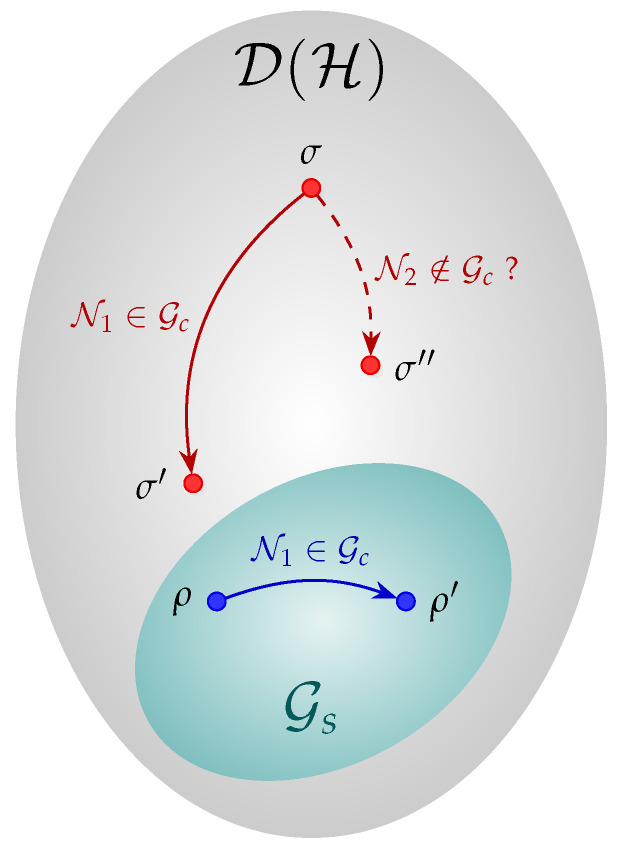
Visual representation of the set of Gaussian states and the action of Gaussian and nG quantum channels relative to reducing the state nG. Every state in $G_{s}$ has $\delta_{nG}=0$, and the action of a Gaussian channel for any state outside $G_{s}$ reduces its nG. The same is not necessarily true for nG channels.

In an nG-QRT, the QRE-based non-Gaussianity measure is a resource quantifying function and carries some operational meaning in its properties. In such a resource theory, Gaussian states and Gaussian operations are free [11]. Property (ii) implies that no free operation should increase the amount or resource of a quantum state. Then, one way of looking at the questioning about whether an nG quantum channel can reduce the non-Gaussianity of nG states can be rephrased as “can property (ii) be extended to nG quantum channels?”. This idea is illustrated in Figure 1, where $N_{1}$ is a Gaussian channel and, as such, maps Gaussian states into Gaussian states ($\hat{\rho }$ and ${\hat{\rho }}^{\prime }$) and reduces the non-Gaussianity of $\hat{\sigma }\notin G_{s}$. For the sake of simplicity, the non-Gaussianity of a quantum state is illustrated as the distance from the set $G_{s}$ and should not be taken formally. The question remaining is whether there is any nG channel $N_{2}$ still bringing states closer to the Gaussian sector.

Now, consider the following setup: Let $\left\{X_{n}\right\}$ be a sequence of random variables (with corresponding alphabet $X_{n}$ and probability mass function $p_{X_{n}}\left(x\right)$) such that it converges in distribution to a complex Gaussian density, that is, $X_{n}\overset{D}{\to }X_{G}∼N_{C}\left(0,\bar{m}\right)$. For this work, we consider $X_{n}$ to be distributed symmetrically on the complex plane, i.e., $p_{X_{n}}\left(x\right)=p_{X_{n}}\left(-x\right)$ for any $x\in X_{n}$. For each $X_{n}$, define the ensemble of coherent states $\{\left|x\rangle ,p_{X_{n}}\left(x\right)\right\}$ and the corresponding mixed state ${\hat{\rho }}_{X_{n}}=\sum_{x\in X_{n}}p_{X_{n}}\left(x\right)|x\rangle \langle x|$. Such an ensemble can represent what a transmitter (Alice) can send to the receiver (Bob) through a quantum channel $N_{A\to B}$ in a CV-QKD protocol with discrete modulation (DM), where the modulation format is represented by the random variable $X_{n}$.

By considering a mixture of coherent states that is induced by a random variable taken from a sequence that converges to a Gaussian distribution, one can prove the following statements [15]:

(2) $$\begin{array}{cc} \lim_{n\to \infty }\delta_{vN}\left({\hat{\rho }}_{X_{n}}\right) & =0 \end{array}$$

(3) $$\begin{array}{cc} \lim_{n\to \infty }\underset{N_{A\to B}}{\inf}\{\delta_{vN}\left(N_{A\to B}\left({\hat{\rho }}_{X_{n}}\right)\right)\} & =0, \end{array}$$

where the infimum must be computed for all quantum channels $N_{A\to B}$ compatible with the statistics observed at the output up to the second moment (We point out that in [15], the main problem was the analysis of the role of non-Gaussianity in security proofs of continuous-variable quantum key distribution protocols using discrete modulation of coherent states. That is why it is relevant to restrict the quantum channels to the ones matching the first and second statistical moments at the reception when defining the infimum in Equation (3)). It is important to note that the quantum channel that minimizes the QRE-nG function in Equation (3) does not need to be Gaussian. Consider an arbitrary $N\in G_{c}$ and let $N^{*}\in Q$ be the quantum channel for which the infimum in Equation (3) is achieved for some *n*. Then, using the monotonicity of $\delta_{vN}$, the following inequality holds:

(4) $$\delta_{vN}\left({\hat{\rho }}_{X_{n}}\right)\geq \delta_{vN}\left(N\left({\hat{\rho }}_{X_{n}}\right)\right)\geq \delta_{vN}\left(N^{*}\left({\hat{\rho }}_{X_{n}}\right)\right),$$

and by adding the convergence $X_{n}\overset{D}{\to }X_{G}∼N_{C}\left(0,\bar{m}\right)$, one has that

(5) $$\lim_{n\to \infty }\delta_{vN}\left(N^{*}\left({\hat{\rho }}_{X_{n}}\right)\right)\leq \lim_{n\to \infty }\delta_{vN}\left({\hat{\rho }}_{X_{n}}\right)=0.$$

Besides the guarantee of small non-Gaussianity before and after the quantum channel for sufficiently large *n* whenever $X_{n}\to X_{G}$, there is no constraint for $N^{*}$ to be Gaussian. The question of extending property (ii) of $\delta_{vN}$ gains operational importance in understanding which conditions a quantum channel has to satisfy for one to be able to compute the minimal output non-Gaussianity, given the input state and the data statistics up to the second moment. As will be discussed in the following sections, this minimal non-Gaussianity quantity can be directly linked to the problem of accessing the security of CV-QKD protocols with discrete (nG) modulation.

## 3. Quantum Relative Entropy Monotonicity Under Non-Gaussian Quantum Channels

In this section, we will develop the conditions under which an nG quantum channel maintains the monotonic property of the QRE non-Gaussianity and provide numerical results for a class of mixtures of quantum states that are of practical interest for CV-QKD protocols. The first step is to prove the following lemma:

**Lemma** **1.** *Let N be a quantum channel, let $\hat{\rho }$ be an arbitrary quantum state, and define*

(6) $$\Delta \left(N,\hat{\rho }\right)=\mathrm{tr}[N\left(\hat{\rho }\right)\left(\log N{\left(\hat{\rho }\right)}^{G}-\log N\left({\hat{\rho }}^{G}\right)\right)].$$

*If $N\in G_{c}$ then $\Delta (N,\hat{\rho })=0$ for any quantum state $\hat{\rho }$*

**Proof.**  If N is a Gaussian channel and Γ is the covariance matrix of an arbitrary quantum state $\hat{\rho }$, then $\Gamma \left(\hat{\rho }\right)=\Gamma \left({\hat{\rho }}^{G}\right)$ and $\Gamma \overset{N}{\to }\Gamma^{\prime }$. This means that $\Gamma \left(N{\left(\hat{\rho }\right)}^{G}\right)=\Gamma \left(N\left({\hat{\rho }}^{G}\right)\right)=\Gamma^{\prime }$. Since the first moment will follow in the same way, $N\left({\hat{\rho }}^{G}\right)=N{\left(\hat{\rho }\right)}^{G}$ for any $\hat{\rho }\in D(H)$, and then $\Delta (N,\hat{\rho })=0$ for arbitrary $\hat{\rho }$. □

This result gives a sufficient condition to classify a quantum channel concerning its non-Gaussianity: if it is verified that $\Delta (N,\hat{\rho })\neq 0$ for some quantum state $\hat{\rho }$, then N is nG. Now, define the set of quantum channels for which Δ≥0, $F=\{N\in Q:\Delta (N,\hat{\rho })\geq 0\;\forall \;\hat{\rho }\in D(H)\}$. Then, $G_{c}\subset F$ and this allows us to propose the following statement:

**Theorem** **1.** *If N∈F, then $\delta_{vN}\left(N\left(\hat{\rho }\right)\right)\leq \delta_{vN}\left(\hat{\rho }\right)$ for any $\hat{\rho }\in D(H)$.*

**Proof.**  Let $\hat{\rho }$ and N be as in the setup. From quantum relative entropy contractivity under quantum channels, one gets

(7) $$\begin{array}{cc} \delta_{vN}\left(\hat{\rho }\right) & \overset{\left(a\right)}{=}S(\hat{\rho }||{\hat{\rho }}^{G}) \end{array}$$

(8) $$\begin{array}{cc} \; & \overset{\left(b\right)}{\geq }S(N\left(\hat{\rho }\right)||N\left({\hat{\rho }}^{G}\right)) \end{array}$$

(9) $$\begin{array}{cc} \; & \overset{\left(c\right)}{=}\mathrm{tr}[N\left(\hat{\rho }\right)\left(\log N\left(\hat{\rho }\right)-\log N\left({\hat{\rho }}^{G}\right)\right)]+\mathrm{tr}[\left(N\left(\hat{\rho }\right)-N{\left(\hat{\rho }\right)}^{G}\right)\log N{\left(\hat{\rho }\right)}^{G}] \end{array}$$

(10) $$\begin{array}{cc} \; & \overset{\left(d\right)}{=}S\left(N{\left(\hat{\rho }\right)}^{G}\right)-S\left(N\left(\hat{\rho }\right)\right)+\Delta \left(N,\hat{\rho }\right) \end{array}$$

(11) $$\begin{array}{cc} \; & \overset{\left(e\right)}{=}{S(N\left(\hat{\rho }\right)||N\left(\hat{\rho }\right)}^{G})+\Delta \left(N,\hat{\rho }\right) \end{array}$$

(12) $$\begin{array}{cc} \; & \overset{\left(f\right)}{\geq }\delta_{vN}\left(N\left(\hat{\rho }\right)\right), \end{array}$$

where (a) comes from the definition of $\delta_{vN}$, (b) from the monotonicity of quantum relative entropy [16], (c) uses the fact that $\mathrm{tr}[\left(\hat{\sigma }-{\hat{\sigma }}^{G}\right)\log\left({\hat{\sigma }}^{G}\right)]=0$ for arbitrary $\hat{\sigma }$ [17], (d) applies the definition in Lemma 1, (e) follows from Definition 1, and (f) holds because N and $\hat{\rho }$ were chosen such that $\Delta (N,\hat{\rho })\geq 0$. □

Theorem 1 provides an interpretation to the quantity $\Delta (N,\hat{\rho })$, with its non-negativity being a necessary condition for a channel to reduce the non-Gaussianity character of a quantum state. In other words, the $\delta_{vN}$ non-increasing property was extended to nG quantum channels satisfying the conditions of Theorem 1.

However, the specification of F may have been too broad by demanding $\Delta (N,\hat{\rho })$ to be non-negative for all quantum states in the system, and we cannot affirm whether F∖G={⌀} or not. A relaxation in this condition can be achieved by considering only a specific set of quantum states, which we chose to be the states relevant to DM-CVQKD protocols, and can help describe a set of QRE-nG non-increasing quantum channels.

Let $S_{\bar{n}}=\left\{\hat{\sigma }\in D\left(H\right):\hat{\sigma }=\sum_{x\in X_{n}}p\left(x\right){\hat{\rho }}^{th}\left(x,\bar{n}\right)\right\}$ with $X_{n}$ being a discrete symmetric random variable and ${\hat{\rho }}^{th}\left(x,\bar{n}\right)$ be the displaced thermal state with $\bar{n}$ average photons and the first moment x¯=2·(Re{x},Im{x})T. Constellations of coherent states are represented by the set $S_{0}$, and any state in $S_{\bar{n}}$ has a diagonal covariance matrix for any value of $\bar{n}$, which means that its equivalent Gaussian quantum state is a thermal state with the appropriate mean photon number. Then, we can define a relaxed set $F_{\bar{n}}=\{N\in Q:\Delta \left(N,\hat{\rho }\right)\geq 0\;\forall \;\hat{\rho }\in S_{\bar{n}}\}$ such that the QRE-nG of any quantum state in $S_{\bar{n}}$ is non-increasing under the action of any channel in $F_{\bar{n}}$. Also, we have $G_{c}\subset F\subset F_{\bar{n}}$ for any $\bar{n}$. The states in $S_{\bar{n}}$ are relevant for the QKD setup because they represent the mixed states output by a noisy modulation device with modulation noise $\bar{n}$. We can affirm the following proposition:

**Proposition** **1.** *$F_{0}\setminus G\neq \{⌀\}$.*

**Proof.**  Take the phase diffusion process described in Appendix A and represent it by $N_{\kappa }$, which is the model of a non-Gaussian evolution of a quantum system. It is known that the QRE-nG of coherent states under phase diffusion increases with the diffusion parameter κ. In Appendix A, it is shown that for any $\hat{\rho }\in S_{0}$, $\bar{x}\left(\hat{\rho }\right)=\bar{x}\left(N_{\kappa }\left(\hat{\rho }\right)\right)=0$ and $\Gamma \left(\hat{\rho }\right)=\Gamma (N_{\kappa }\left(\hat{\rho }\right))$, which implies that ${\hat{\rho }}^{G}=N_{\kappa }{\left(\hat{\rho }\right)}^{G}$. That is, the phase diffusion process does not modify the first and second statistical moments of appropriate mixtures of coherent states. In addition, it does not affect thermal states, meaning that $N_{\kappa }\left({\hat{\rho }}^{G}\right)={\hat{\rho }}^{G}$. We conclude that $N_{\kappa }\left({\hat{\rho }}^{G}\right)=N_{\kappa }{\left(\hat{\rho }\right)}^{G}$, which results in $\Delta (N_{\kappa },\hat{\rho })=0$ for any state in $S_{0}$ and then $N_{\kappa }\in F_{0}\setminus G\neq \{⌀\}$. □

**Figure 2:**
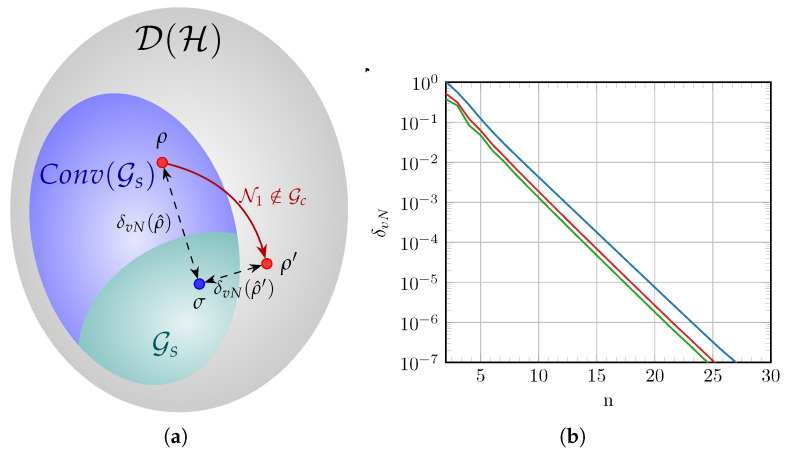
(**a**) Visual representation of the action of an nG quantum channel on an nG quantum state according to Proposition 1. The set $Conv(G_{s})$ is the convex hull of $G_{s}$. (**b**) Values of the QRE-nG for the GH-QAM constellation with *m* points per quadrature under a phase diffusion process with fixed modulation variance $\bar{m}=2.5$ and increasing constellation size. The upper line (blue) corresponds to the constellation nG before the channel and in the constellation under the process with parameters κ=0.15 and κ=∞, respectively.

We conjecture that Proposition 1 can be extended to other values of $\bar{n}$ different from zero, although we have not yet worked out the proof. A graphical representation of the action of the phase diffusion channel is given in Figure 2a. The states in $S_{0}$ are convex mixtures of Gaussian states which, by linearity of the quantum channel, are mapped to a convex mixture of nG states ${\hat{\rho }}^{\prime }=N_{\kappa }\left(\hat{\rho }\right)$. Since the phase diffusion process does not modify the covariance matrix of the states in $S_{0}$, both $\hat{\rho }$ and ${\hat{\rho }}^{\prime }$ have the same covariance matrix and the same Gaussian equivalent state, represented by $\hat{\sigma }$ in Figure 2a. The consequence of Proposition 1 is that ${\hat{\rho }}^{\prime }$ is closer to $\hat{\sigma }$ than $\hat{\rho }$ in the sense of QRE.

To illustrate how an nG channel can reduce the non-Gaussianity of an ensemble of coherent states, consider QAM-like (Quadrature Amplitude Modulation) constellations of coherent states, where each quadrature follows a Gauss–Hermite distribution. Such constellations are known to converge exponentially to the capacity of the additive white Gaussian noise (AWGN) channel in classical communication scenarios [18].

The Gauss–Hermite constellation is constructed by taking the *n*-th Hermite polynomial, defined via the derivatives of the standard Gaussian probability density function $p_{X}\left(x\right)$:

(13) $$H_{n}\left(x\right)=\frac{{\left(-1\right)}^{n}}{p_{X}\left(x\right)}\frac{d^{n}p_{X}\left(x\right)}{dx^{n}}.$$

This polynomial has *n* distinct real roots, denoted by the set $X_{n}$, which determine the constellation points. Each root $x_{i,n}$ is associated with a weight (interpreted as a probability) given by the following:

(14) $$w_{i,n}=\frac{(n-1)!}{nH_{n-1}^{2}\left(x_{i,n}\right)}.$$

**Figure 3:**
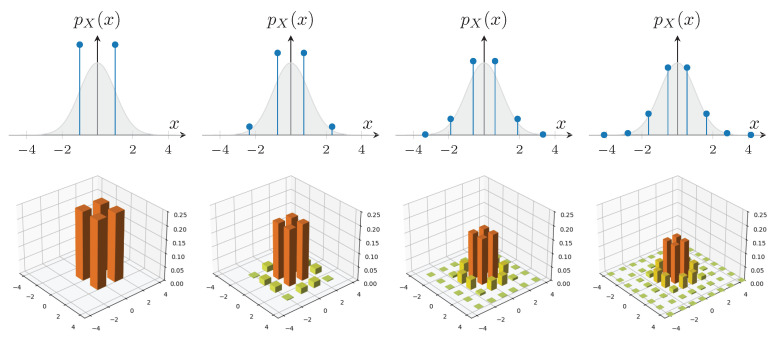
Constellations resulting from the Hermite polynomials of Equation (13) with n∈{2,4,6,8}. On top, the roots and weights of $H_{n}\left(x\right)$ form the probability distribution $p_{X}\left(x\right)$. At the bottom, the corresponding QAM-like constellations obtained by the Cartesian product are shown.

Examples of these constellations are shown in Figure 3. The top row displays the roots and associated weights of $H_{n}\left(x\right)$ for n=2,4,6,8, representing one-dimensional constellations along a single quadrature. As *n* increases, the point distribution increasingly resembles a Gaussian profile. The bottom row shows the resulting QAM-like two-dimensional constellations formed by taking the Cartesian product of two independent one-dimensional Gauss–Hermite constellations, corresponding to statistically independent quadratures.

Figure 2b shows the numerical results for the computation of the QRE-based non-Gaussianity of QAM-like constellations, with each axis following a Gauss–Hermite distribution and undergoing a phase diffusion process. The upper blue line corresponds to the QRE-nG measure for the constellations before the phase diffusion process takes place. The lines below represent the calculated $\delta_{vN}\left(N_{\kappa }\left({\hat{\rho }}_{X_{n}}\right)\right)$ for κ=0.15 (red line) and κ=∞ (green line), the latter having the effect of total decoherence in the mixture of coherent states, which destroys the off-diagonal elements in the density matrix. The occasional observed fluctuations near the left end of the plot (low values of *n*) for the red and green lines can be attributed to the action of the non-Gaussian process, which can map the ensemble of input states to output states with a slightly varying non-Gaussianity reducing rate when the constellation size is still small. After approximately seven states per quadrature, the output-state non-Gaussianity transitions to a clear exponential decay as expected.

As expected, since the Gauss–Hermite distribution converges exponentially to a Gaussian shape, the QRE-based non-Gaussianity also decreases exponentially as the constellation grows. Concerning the action of $N_{\kappa }$, two points can be highlighted: First, the bigger the diffusion parameter κ gets, the lower the constellation non-Gaussianity after the channel is, which is related to the counterintuitive fact that $N_{\kappa }$ maps to a convex mixture of nG states that is more Gaussian than the mixed state before it. The second is that the effect of undergoing the nG evolution becomes less significant (in the sense of reducing the state non-Gaussianity) as the mixture becomes more Gaussian-like. For example, if we compute the input–output non-Gaussianity difference $\delta_{vN}\left({\hat{\rho }}_{X_{n}}\right)-\delta_{vN}\left(N_{\kappa }\left({\hat{\rho }}_{X_{n}}\right)\right)$, with κ=0.15, one has that it is $\approx 2.4\times 10^{-3}$ for n=10 and goes to $\approx 4.8\times 10^{-6}$ for n=20. This is somehow expected because thermal states commute with the Krauss operators representing the phase diffusion evolution, meaning that as ${\hat{\rho }}_{X_{n}}$ becomes “more Gaussian”, $N_{\kappa }$ has less effect on it.

## 4. Discussion: Application to QKD Protocols

The basic operation of a QKD protocol can be divided into four main stages: (i) quantum state preparation, transmission, and detection; (ii) classical parameter estimation; (iii) information reconciliation; and (iv) privacy amplification. A QKD protocol that applies discrete modulation—i.e., uses a constellation of symbols—to the quadratures of continuous-variable quantum systems, such as coherent states, is referred to as a DM-CVQKD protocol. The coherent states are prepared with amplitudes drawn from a random variable, transmitted and detected by either a single- or a double-homodyne receiver. The resulting shared sequences—the input data used to modulate the coherent states and the detection outcomes—are called the raw key and should be used to distill a secret random sequence given that they present enough correlation, which is evaluated by the parameter estimation stage under a specific security model. After that, error correction is performed by some information reconciliation protocol, and the eavesdropper information is removed by privacy amplification.

Finding the worst-case eavesdropping strategy is a crucial step towards proving the security of a QKD protocol. For the class of Gaussian-modulated protocols with continuous variables, such as the GG02, the no-switching, and the unidimensional protocols [19,20,21], the optimality of Gaussian attacks is a pivotal result simplifying the security analysis: if the protocol is based on a Gaussian modulation of coherent states (this means that Alice will transmit coherent states whose amplitudes are drawn from a circular Gaussian distribution, or equivalently, the amplitude on each quadrature is drawn from independent and equally distributed Gaussian random variables), the best Eve can do is to perform the “entangling cloner attack”, which is equivalent to a Gaussian quantum channel with transmittance τ and excess noise ξ [22,23,24,25]. The consequence is that Alice and Bob can safely assume the Gaussian channel model in the security analysis.

The problem completely changes when Alice applies a discrete modulation. In this case, it is not guaranteed that Gaussian attacks are optimal, and the security analysis must include nG quantum channels compatible with the parameters observed in the classical data for computing the eavesdropper’s information [12]. This means that Alice and Bob must estimate the channel parameters τ and ξ using their classical data and compute the eavesdropper information for the *worst-case scenario*, considering any type of quantum channel resulting in the estimated parameters. This reduces to the known Devetak–Winter formula for the secret key rate in the asymptotic scenario:

(15) $$K=\beta I\left(A;B\right)-\underset{N\in Q}{\sup}\chi \left(B;E\right),$$

where β is the efficiency of information reconciliation, I(A;B) is the classical mutual information of Alice and Bob’s raw keys, and χ(E,B) is the eavesdropper’s accessible information during quantum communication considering reverse reconciliation. In this type of reconciliation, error correction is performed by taking as reference the receiver’s sequence so that Alice has to modify her sequence towards Bob’s one, differently from a classical communication task. This maneuver allows the protocol to be able to establish secret keys beyond the 3 dB loss limit.

**Figure 4:**
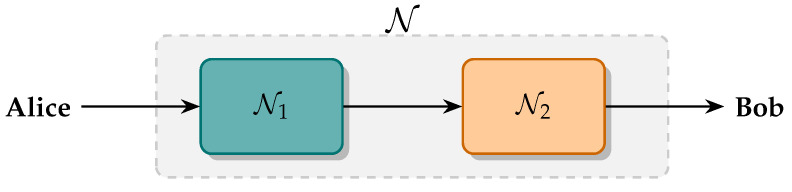
Representation of the proposed quantum channel decomposition. The channel N connecting Alice and Bob is split into a thermal-loss part $N_{1}$ and a non-Gaussian evolution $N_{2}$. Alice and Bob reconstruct the covariance matrix by estimating the parameters yielded by $N_{1}$. Any security analysis that goes beyond considering Gaussian channels should then quantify the effect of $N_{2}$ during quantum communication.

In Section 3, it was shown that the phase diffusion process preserves the monotone property of the QRE-nG when restricted to an appropriate set of quantum states. Additionally, it does not modify the covariance matrix of the input state. Both statements can be used in the analysis of DM-CVQKD protocols by proposing the following arrangement to decompose the quantum channels considered in the security analysis. Denote by T the set of quantum channels that preserve the first and second moments of any quantum state in $S_{\bar{0}}$. Without loss of generality, assume that Alice and Bob are linked by $N=N_{2}\circ N_{1}$, as depicted in Figure 4, where $N_{1}\in T$ and $N_{2}\in G_{s}$ is a thermal-loss channel with transmittance τ and excess noise ξ.

The idea here is to decompose the quantum channel into two parts, the Gaussian $N_{2}$ yielding physical parameters in a practical deployment of a QKD protocol, raising the observed parameters τ and ξ, and $N_{1}$, which does not modify the covariance matrix (and then does not affect the parameter estimation) but is responsible for non-Gaussian interactions giving more information to the eavesdropper. Such decomposition may allow more accurate lower bounds to the secret key rate by performing a more efficient computation of the eavesdropper’s information in security analysis.

Take as an example the security analysis of [12]. Its main objective was to analytically deduce a correction factor for the covariance matrix describing the bipartite state on a DM-CVQKD protocol. When restricting the problem to a linear Gaussian channel, the corrected off-diagonal therm Z(τ,ξ) of Γ is

(16) $$Z\left(\tau ,\xi \right)=2\sqrt{T}\mathrm{tr}\left({\hat{\rho }}^{1/2}\hat{a}{\hat{\rho }}^{1/2}a^{†}\right)-\sqrt{2T\xi w},$$

with $\hat{a}$ and ${\hat{a}}^{†}$ being the field annihilation and creation operators, respectively; $w:=\sum_{k}p_{k}(\langle \alpha_{k}|{\hat{a}}_{\rho }^{†}{\hat{a}}_{\rho }|\alpha_{k}\rangle -{\left|\langle \alpha_{k}|{\hat{a}}_{\rho }|\alpha_{k}\rangle \right|}^{2})$ and ${\hat{a}}_{\rho }={\hat{\rho }}^{1/2}\hat{a}{\hat{\rho }}^{-1/2}a^{†}$. Such a correction factor works as a penalty for describing an nG state with just its second moment. One could extend the analysis by relating its results for arbitrary channels to the minimization of the output-state QRE non-Gaussianity measure, which is also related to a penalty due to nG modulation [15], subject to the empirical constraints estimated on a practical protocol: channel parameters and the expected covariance. This yields a rigorous lower bound on the protocol by estimating the effects of the channel’s non-Gaussianity, without the need for knowing upfront which nG quantum channel connects Alice and Bob. The resulting operational framework fits naturally into DM-CVQKD protocols where these quantities are already monitored, enabling practical application of our theoretical results and facilitating tighter security bounds when non-Gaussianity is empirically small.

## 5. Conclusions

We explored the conditions under which a non-Gaussian quantum channel reduces the amount of non-Gaussianity of a quantum channel using the quantum relative entropy as a quantifying function. We proposed the functional $\Delta (N,\hat{\rho })$ that can be used to classify the channel N as Gaussian or non-Gaussian. Based on $\Delta (N,\hat{\rho })$, we developed a condition under which a non-Gaussian channel reduces the non-Gaussianity of its input states. This result extends the monotone decreasing property of the quantum relative entropy-based non-Gaussianity measure to outside the Gaussian sector of quantum operations.

The characterization of non-Gaussian channels that reduce the non-Gaussianity of input states was used to establish a link between the security analysis of CVQKD protocols and the class of non-Gaussianity-reducing quantum channels. A decomposition of the general channels considered in the security analysis of CVQKD was proposed, with operational implications. It is still an open problem how this decomposition can improve the secret key rate bounds computed with today’s security analysis framework. In addition, it may be possible that this decomposition can be used to improve parameter estimation procedures.

Future work can also be concentrated on generalizing Proposition 1 and developing the properties of $\Delta (N,\hat{\rho })$. In addition, it should be noted that the difference $S(\hat{\rho }||{\hat{\rho }}^{G})-S(N\left(\hat{\rho }\right)||N\left({\hat{\rho }}^{G}\right))$ (see the proof of Theorem 1) is related to state recovery maps (Petz recovery maps), which are maps that can recover the state that suffered some physical evolution. Such recovery maps can be extended to quantum systems in infinite dimensions [26] and may have connections with the “production of non-Gaussianity” and with CVQKD security analysis.

## Data Availability

The original contributions presented in this study are included in the article. Further inquiries can be directed to the corresponding author.

## Appendix A. The Phase Diffusion Process

One of the most harmful noisy processes in quantum information is one that provokes decoherence, where quantum states lose their “quantumness”—full decohered states reduce to mixtures of orthogonal states which can be perfectly distinguished. Some open-system processes result in random changes in the relative phase of the states that are in superposition in the main quantum system. Such a relative phase fluctuation results in a loss of coherence and is called phase damping or phase diffusion [27].

The single-mode evolution of the system under such a process can be described by the master equation [11,28]:

(A1) $$\frac{d}{dt}\hat{\rho }=\Gamma L[{\hat{a}}^{†}\hat{a}]\hat{\rho },$$

where $L\left[\hat{O}\right]\hat{\rho }=2{\hat{O}}^{†}\hat{\rho }\hat{O}-{\hat{O}}^{†}\hat{O}\hat{\rho }-\hat{\rho }{\hat{O}}^{†}\hat{O}$, or as the Hamiltonian of a harmonic oscillator open to an *N* mode environment [27]:

(A2) $$H=\hbar \omega {\hat{a}}^{†}\hat{a}+\hbar \sum_{i=1}^{N}\omega_{i}{\hat{a}}_{i}^{†}{\hat{a}}_{i}+\hbar \sum_{i=1}^{N}\chi_{i}{\hat{a}}^{†}\hat{a}\left({\hat{a}}_{i}+{\hat{a}}_{i}^{†}\right),$$

where $\hat{a}$ and ${\hat{a}}^{†}$ are the annihilation and creation operators of the main system with frequency ω, and ${\hat{a}}_{i}$ and ${\hat{a}}_{i}^{†}$ refer to the *i*th environment system with frequency $\omega_{i}$. The quantity $\chi_{i}$ represents a coupling parameter between the main system and the *i*th environment mode.

This non-Gaussian evolution of a quantum state is an important source of noise in optical communication links. Its Krauss operator set $\left\{P_{k}\left(t\right)\right\}$, 0≤k≤∞ has elements

(A3) $$P_{k}\left(t\right)=\sum_{n=0}^{\infty }e^{-\frac{1}{2}n^{2}\lambda^{2}}\sqrt{\frac{{\left(n^{2}\lambda^{2}\right)}^{k}}{k!}}|n\rangle \langle n|$$

where $\lambda =t\sqrt{\Lambda }$ and $\Lambda =\sum_{i}\chi_{i}^{2}\sqrt{1-e^{-n^{2}\lambda^{2}}}$.

Regarding the effect of phase diffusion on Gaussian states, the thermal state with average photon number $\bar{n}$, ${\hat{\rho }}^{th}\left(\bar{n}\right)$, is invariant under the phase diffusion process state, since both $\bar{n}$ and $P_{k}\left(t\right)$ are diagonal in the same basis. For coherent states, it is well known that they are sensitive to relative phase fluctuations: for a coherent state |α〉 with arbitrary α, one has

(A4) $$\begin{array}{cc} N_{\kappa }\left(|\alpha \rangle \langle \alpha |\right)= & e^{-{\left|\alpha \right|}^{2}}\sum_{m,n=0}^{\infty }\exp\{-\kappa^{2}{\left(n-m\right)}^{2}\}\times \frac{\alpha^{n}\alpha^{*m}}{\sqrt{n!m!}}|n\rangle \langle m|, \end{array}$$

which is a non-Gaussian mixed state with $\kappa =\lambda^{2}/2$.

In contrast to thermal states, coherent states suffer from decoherence in a phase diffusion evolution, with the off-diagonal elements of the density operator being more affected as the noise parameter κ becomes larger.

Now, consider the mixtures of coherent states as defined in Section 3 by the set $S_{0}$. Denote a constellation by the set of complex amplitudes $\left\{\alpha_{i}\right\}$ with associated probabilities $\left\{p_{i}\right\}$. Also, by symmetry, $\sum p_{i}\alpha_{i}=0$, and let $\bar{m}=\sum p_{i}{\left|\alpha_{i}\right|}^{2}$ be the modulation variance. Represent the constellation of coherent state by $\hat{\rho }=\sum_{i}p_{i}|\alpha_{i}\rangle \langle \alpha_{i}|$, and denote ${\hat{\rho }}_{\kappa }=N_{\kappa }\left(\hat{\rho }\right)$. Due to the linearity of the channel, we use (A4) directly and have

(A5) $${\hat{\rho }}_{\kappa }=\sum_{m,n=0}^{\infty }e^{-\kappa^{2}{\left(n-m\right)}^{2}}\sum_{i=0}^{N}p_{i}e^{-|\alpha_{i}{|}^{2}}\frac{\alpha_{i}^{n}\alpha_{i}^{*m}}{\sqrt{n!m!}}|n\rangle \langle m|.$$

Clearly, as each coherent state is transformed into an nG mixed state, the resultant mixture will also be nG, although in contrast to a single coherent state, a symmetric convex mixture has the first and second moments invariant under phase diffusion, that is,

(A6) $$\mathrm{tr}\left(\hat{q}{\hat{\rho }}_{\kappa }\right)=\mathrm{tr}\left(\hat{p}{\hat{\rho }}_{\kappa }\right)=0,$$

and

(A7) $$\begin{array}{c} \Gamma =\left(\begin{array}{cc} 1+2\bar{m} & 0\\ 0 & 1+2\bar{m} \end{array}\right) \end{array}$$

so that $\Gamma \left({\hat{\rho }}_{\kappa }\right)=\Gamma \left(\hat{\rho }\right)$.
